# Histopathologic characteristics of biopsies from dogs undergoing surgery with concurrent gross splenic and hepatic masses: 125 cases (2012–2016)

**DOI:** 10.1186/s13104-018-3220-1

**Published:** 2018-02-13

**Authors:** Fernando J. Leyva, Catherine A. Loughin, Curtis W. Dewey, Dominic J. Marino, Meredith Akerman, Martin L. Lesser

**Affiliations:** 1 Department of Surgery, Long Island Veterinary Specialists, Plainview, NY 11803 USA; 2 Department of Clinical Sciences, College of Veterinary Medicine, Cornell University, Ithaca, NY 14853 USA; 3 Biostatistics Unit, Feinstein Institute for Medical Research, Northwell Health, 350 Community Dr, Manhasset, NY 11030 USA

**Keywords:** Canine biopsies, Dog biopsies, Splenic mass, Hepatic mass, Histopathology, Hemangiosarcoma

## Abstract

**Objective:**

To investigate the histopathologic characteristics of concurrent splenic and liver masses in dogs undergoing splenectomy and liver mass biopsy/resection. Medical records of 125 client-owned dogs found to have splenic mass or masses and a liver mass or masses during surgery were examined. Signalment (age, sex, breed), body weight, and results of histopathology were recorded for all dogs.

**Results:**

Twenty-seven percent (34/125) of the dogs in this study had no evidence of malignancy in either the liver or the spleen. Sixty of 125 dogs (48.0%) had malignancy in the spleen and liver, and 56 (56/60, 93.3%) of those dogs had the same malignancy in both organs. Signalment was similar to that in other reports of splenic pathology. In this clinical population of dogs, 27% of dogs with concurrent gross splenic and liver masses discovered intraoperatively had benign lesions in both locations and therefore had a favorable prognosis.

**Electronic supplementary material:**

The online version of this article (10.1186/s13104-018-3220-1) contains supplementary material, which is available to authorized users.

## Introduction

Splenic masses are frequently encountered in dogs. They may be diagnosed in dogs that present with non-traumatic hemoabdomen or incidentally upon imaging or surgery. Lesions in the spleen may be the result of benign (i.e. lymphoid hyperplasia, hematoma, cyst, abscess, etc.) or malignant (i.e. hemangiosarcoma (HSA), metastatic sarcoma/carcinoma, malignant histiocytosis, lymphosarcoma (LSA), etc.) processes [1–4]. Hemangiosarcoma has been reported by some researchers as the most common splenic mass in dogs [1, 5]; however, others have reported higher prevalence of benign lesions [2–4]. In contrast, the prevalence of benign versus malignant lesions has been well established in dogs with non-traumatic hemoabdomens. The study by Johnson et al. [5] proposed the “law of two-thirds” in dogs with non-traumatic hemoabdomens, in which two-thirds of dogs with splenomegaly have neoplasia and two-thirds of these had hemangiosarcoma.

Similar to splenic masses, hepatic masses may be benign (i.e. nodular hyperplasia (NH), extramedullary hematopoiesis, cyst, abscess, hematoma, etc.) or malignant (i.e. hepatocellular carcinoma (HCC), LSA, malignant histiocytosis, HSA, metastatic carcinoma/sarcoma, etc.) [6–9]. The presence of a concurrent hepatic mass or masses (HM) with a splenic mass or masses (SM) has been reported to be associated with a metastatic disease process [10, 11]. Hemangiosarcoma and other malignant splenic neoplasms have been reported to metastasize to the liver, via splenic venous blood, lymphatic drainage, or transcoelomic metastasis [12, 13]. However, it has also been reported that there is a high prevalence of benign hyperplastic hepatic nodules in dogs with increasing age [14]. The presence of HM, SM, and the potential for gross metastatic disease are important considerations for many clients in determining whether they wish to accept the costs, risks, and morbidity associated with surgery given the possibility of a poor long-term prognosis [15].

The purpose of this retrospective study was to describe the prevalence of benign versus malignant masses in dogs with SM and HM undergoing surgery, and to report patient signalment (age, sex, breed), weight, and histopathological diagnosis of both organs.

## Main text

### Methods

#### Inclusion criteria/case selection

A database search in infinity^1^ of our medical records revealed 369 splenectomies which were performed between January 1, 2012, and January 1, 2016. The medical records of all 369 dogs were reviewed. Only dogs with both SM and HM found at surgery were included in this study. One hundred twenty-five client-owned dogs were identified with confirmed SM and HM at the time of surgery and were included in this study. Data obtained from the medical records included histopathological diagnosis of both splenic and hepatic tissue, signalment (age, sex, breed), and body weight. All surgeries were performed by surgeons (ACVS diplomates) or surgical residents under supervision of ACVS diplomates, after written consent was provided by the owners. Each dog had a splenectomy performed in conjunction with a liver biopsy (via liver lobectomy or incisional biopsy).

#### Histopathologic evaluation

Following splenectomy, each whole spleen and liver sample were fixed in 10% buffered formalin, and were reviewed by board-certified pathologists from a local, outside laboratory (Antech Diagnostics^2^). Sections from microscopic examination were selected from various regions of the spleen and liver samples submitted; generally multiple sections were obtained from the margin of any nodule and adjacent unaffected parenchyma. Microscopic evaluation of multiple selected sections was accomplished from 4- to 6-μm sections of paraffin-embedded tissue mounted on glass microslides and stained with hematoxylin and eosin. Additional stains were performed at the discretion of the pathologist. Based on the microscopic features of the tissues, the lesions were characterized histologically.

#### Statistical analysis

Descriptive statistics (mean, SD, median, minimum, and maximum values for age and weight; frequency and percentage for categorical variables such as sex, breed, benign or malignant lesions, type of lesion) were calculated for the study sample. A cross-tabulation of spleen and liver status (benign or malignant) was constructed to determine the percent of dogs with benign splenic lesions and benign liver lesions, the percent of dogs with benign splenic lesions and malignant liver lesions, the percent of dogs with malignant splenic lesions and malignant liver lesions, and the percent of dogs with malignant splenic lesions and benign liver lesions.

Inferential statistics were also conducted for the study sample. After testing for equality of variance by Levene’s test, a series of independent-samples t tests were conducted for age and weight. A series of Chi squared analyses for patient sex was also conducted. Values of *P* ≤ 0.05 were considered significant for all analyses. All analyses were performed using SAS version 9.4.^3^

### Results

There were 125 dogs included in this study. Mean age was 10.1 years (range 4–15 years). There were one female intact, 59 female spayed, 11 male intact, and 54 male neutered. Mean weight was 27.8 kg (range 4–58.3 kg). There were 42 mixed breed dogs; 24 Golden R etrievers; 7 Labrador Retrievers; 6 German Shepherd Dogs; 4 Beagles; 3 each Cocker Spaniels, Rottweilers, and Yorkshire Terriers; 2 each Australian Shepherd, Cane Corso, English Springer Spaniel, and Pekingese; and 1 each of 25 other breeds. There were no significant differences in age, weight, or sex between dogs with malignant lesions versus those with concurrent benign lesions. The data generated and analyzed, examining these associations, are summarized in Additional file 1.

Of the 125 dogs, 42 (33.6%) had benign SM and 83 (66.4%) had malignant SM. One of the dogs had two malignant SM. The most common malignant splenic lesion was HSA (69/84, 82.1%). The most common benign splenic lesion was NH (27/62, 43.5%). Sixty-seven dogs (53.6%) had malignant HM. The most common malignant hepatic lesion was HSA (51/68, 75%). The most common benign hepatic lesion was NH (26/69, 37.7%). All biopsy results and number of dogs per type (benign versus malignant) are summarized in Tables 1 and 2.

**Table 1:** Summarization of histopathology results for spleen and liver biopsies

Splenic biopsies obtained in the study	No. (% of total)	No. (% of 125 dogs)
*Malignant splenic lesions*		
Hemangiosarcoma	69 (82.1)	69 (55.2)
Leiomyosarcoma	4 (4.8)	4 (3.2)
Histiocytic sarcoma	3 (3.6)	3 (2.4)
Undifferentiated sarcoma	2 (2.4)	2 (1.6)
Anaplastic spindle cell sarcoma	1 (1.2)	1 (0.8)
Stromal sarcoma	1 (1.2)	1 (0.8)
Osteosarcoma	1 (1.2)	1 (0.8)
Mast cell tumor	1 (1.2)	1 (0.8)
Liposarcoma	1 (1.2)	1 (0.8)
Lymphosarcoma	1 (1.2)	1 (0.8)
Total No. malignant splenic lesions (%)	84 (100)^a^	84 (67.2)^a^
*Benign splenic lesions*		
Nodular hyperplasia	27 (43.5)	27 (21.6)
Hematoma	11 (17.7)	11 (8.8)
Infarct	5 (8.1)	5 (4)
Extramedullary hematopoiesis	4 (6.5)	4 (3.2)
Myelolipoma	3 (4.8)	3 (2.4)
Plaques	3 (4.8)	3 (2.4)
Necrosis	2 (3.2)	2 (1.6)
Splenitis	2 (3.2)	2 (1.6)
Congestion	2 (3.2)	2 (1.6)
Lipoma	1 (1.6)	1 (0.8)
Lymphoid hyperplasia	1 (1.6)	1 (0.8)
Hemosiderosis	1 (1.6)	1 (0.8)
Total No. benign splenic lesions (%)	62 (100)^a^	62 (49.6)^a^

Liver biopsies obtained in the study	No. (% of total)	No. (% of 125 dogs)
*Malignant liver lesions*		
Hemangiosarcoma	51 (75)	51 (40.8)
Hepatocellular carcinoma	4 (5.9)	4 (3.2)
Histiocytic sarcoma	3 (4.4)	3 (2.4)
Leiomyosarcoma	3 (4.4)	3 (2.4)
Lymphosarcoma	2 (2.9)	2 (1.6)
Undifferentiated sarcoma	2 (2.9)	2 (1.6)
Anaplastic spindle cell sarcoma	1 (1.5)	1 (0.8)
Liposarcoma	1 (1.5)	1 (0.8)
Stromal sarcoma	1 (1.5)	1 (0.8)
Total No. malignant liver lesions (%)	68 (100)^a^	68 (54.4)^a^
*Benign liver lesions*		
Nodular hyperplasia	26 (37.7)	26 (20.8)
Hydrophic vacuolar hepatopathy	16 (23.2)	16 (12.8)
Hepatoma	6 (8.7)	6 (4.8)
Hepatitis	4 (5.8)	4 (3.2)
Cholangiohepatitis	2 (2.9)	2 (1.6)
Fibrosis	2 (2.9)	2 (1.6)
Lymphoplasmacytic hepatitis	2 (2.9)	2 (1.6)
Necrosis	2 (2.9)	2 (1.6)
Cirrhosis	2 (2.9)	2 (1.6)
Extramedullary hematopoiesis	1 (1.4)	1 (0.8)
Hematoma	1 (1.4)	1 (0.8)
Lipogranuloma	1 (1.4)	1 (0.8)
Lymphohistiocytic hepatitis	1 (1.4)	1 (0.8)
Congestion	1 (1.4)	1 (0.8)
Infarct	1 (1.4)	1 (0.8)
Cholestasis	1 (1.4)	1 (0.8)
Total No. benign liver lesions (%)	69 (100)^a^	69 (55.2)^a^

^a^Some dogs had multiple lesions on histopathology, thus absolute numbers and percentages do not add up to 125 and 100% respectively

**Table 2:** Number of dogs per type of splenic mass(es) (SM) and hepatic mass(es) (HM)

	Malignant HM	Benign HM	Sum total of dogs
Malignant SM	60	23	83^a^
Benign SM	8	34	42
Sum total of dogs	68	57	125

^a^One dog had two malignant splenic lesions

**Fig. 1:**
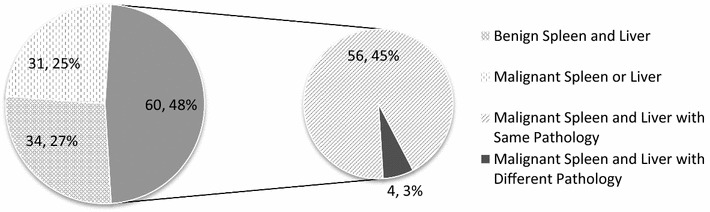
Numbers and percentages of benign and malignant SM and HM (n = 125)

Thirty-four of the dogs (27%) had benign SM and HM. Of the 60 dogs with malignant splenic and liver lesions, 56 (93.3%) had the same malignancy in both organs. Forty-six dogs (76.7%) had HSA in the spleen and the liver, 3 (5%) dogs had leiomyosarcoma (LMS) in both organs, 3 (5%) dogs had histiocytic sarcoma in both organs (one of these dogs additionally had splenic HSA), 1 (1.7%) dog had liposarcoma in both organs, one dog had anaplastic sarcoma in both organs, one dog had undifferentiated sarcoma in both organs, and 1 dog had LSA in both organs. In the four dogs with exclusively different malignancies, one dog had liposarcoma in the spleen and LSA in the liver, one dog had osteosarcoma in the spleen and undifferentiated sarcoma in the liver, one dog had LMS in the spleen and stromal sarcoma in the liver, and the last dog had mast cell tumor in the spleen and HSA in the liver. Seven of the dogs (5.6%) with benign SM had malignant HM, 23 of the dogs (18.4%) with malignant SM had benign HM. The more salient numbers and percentages of dogs with benign and malignant lesions are summarized in Fig. 1.

### Discussion

The identification of concurrent SM and HM poses a conundrum to clinicians. While the incidence of splenic malignancy in SM and non-traumatic hemoabdomens has been well-documented [1, 2, 16, 17], only two studies have looked at the association of HM in dogs with splenic HSA [11, 18]. Our study is the first to report the prevalence of benign versus malignant masses in dogs with concurrent SM and HM undergoing splenectomy. Our study documents that 27% of dogs with SM and HM have benign pathology at both sites and therefore carry a good prognosis. The present study also documents that 48% of dogs with SM and HM have malignant pathology at both sites. Of the 69 dogs in our study that were diagnosed with splenic HSA, 46 (66.7%) had HSA diagnosed in the liver; this is comparable to the results reported by Clendaniel et al. [11] where 29 of 58 (50%) of dogs with gross liver and splenic lesions had splenic HSA metastasis to the liver.

There are a multitude of studies documenting the signalment (age, sex, breed) of dogs with SM [3–5, 15, 18–20]. Multiple studies have found no significant difference in the age of dogs diagnosed with benign versus malignant SM [3, 5, 18, 19]. The present study demonstrates no significant difference in the age of dogs presenting with both SM and HM (10.1 years), versus those of the previously reported studies of SM alone.

Previous studies have found no consistent sex predilection for dogs with benign or malignant SM [5, 20]. The current study similarly did not find a sex predilection between dogs with benign or malignant lesions; however, the large majority of the study population was spayed and castrated. The authors speculate that the reason for the prevalence of gonadectomy was due to cultural and socioeconomic reasons associated with the suburban New York geographical area of our study population.

Our study population was similar to previous reports of SM with respect to breed and weight. In the present study, the dog breeds found to have concurrent SM and HM were comparable to those in previous studies of SM, with mixed breed, Golden Retrievers, Labrador Retrievers, and German Shepherd Dogs being overrepresented [4, 5, 15, 18, 19].

Further prospective studies are warranted and should be directed at quantification and characterization of SM and HM with different imaging modalities and gross examination. Clendaniel et al. [11] previously noted that multiple nodules, dark-colored nodules, and actively bleeding nodules were highly associated with malignancy of the liver. A study utilizing contrast harmonic ultrasonography of splenic masses and liver nodules showed that it was 100% sensitive and specific for differentiating a benign vs malignant processes of the liver; however, the same study found that benign and malignant processes of the spleen were indistinguishable utilizing the same modality [15]. Another study accurately differentiated benign from malignant focal hepatic and splenic lesions in dogs with magnetic resonance imaging; the overall sensitivity and specificity were 100 and 90%, respectively [21]. Cuccovillo et al. [22] noted a positive predictive value of 74% for malignancy when one target lesion was visualized and an 81% positive predictive for malignancy if multiple target lesions were noted in the liver or the spleen on ultrasonography. Future studies should utilize the above mentioned modalities and visual characteristics in series or parallel to better prognosticate cases with SM and HM.

### Conclusions

Our data suggests that nearly 30% of dogs undergoing surgery with both gross hepatic and splenic lesions have a favorable prognosis. While malignant neoplasia is most likely in cases with both SM and HM undergoing splenectomy, benign or treatable causes must be considered possible in each dog. This information should be provided to owners to facilitate making an informed decision of whether or not to pursue surgery after imaging.

## Limitations

Limitations of the study were mainly due to the retrospective nature. Only cases that underwent surgery were included in our study. At our hospital, preoperative abdominal ultrasonography is routinely performed. Given that cases with suspected metastatic disease based on preoperative imaging were likely relayed to owners as such, a sampling bias is probable. The owners of those dogs may have elected euthanasia or palliative therapy at a higher rate than those of other dogs. Thus, our study population may be skewed towards a more favorable prognosis. However, at our hospital we do not recommend euthanasia to owners just on the fact that lesions are noted on both organs and always mention that long-term prognosis would be based on histopathology results.

## Additional file

**Additional file 1.** Supplemental descriptive and inferential statistics. Analyses conducted in order to examine the association between patient age, weight, sex, and malignancies of the spleen and liver.

## Abbreviations

HSA: hemangiosarcoma
LSA: lymphosarcoma
NH: nodular hyperplasia
HCC: hepatocellular carcinoma
HM: hepatic mass or masses
SM: splenic mass or masses
LMS: leiomyosarcoma

## Footnotes

1 Infinity; Henry Schein ImproMed, Oshkosh, WI 54902, United States.

2 Antech Diagnostics; New Hyde Park, NY 11042, United States.

3 SAS version 9.4; SAS Institute Inc., Cary, NC 27513, United States.
