# Supplementary material for: Histopathologic characteristics of biopsies from dogs undergoing surgery with concurrent gross splenic and hepatic masses: 125 cases (2012–2016)
Source: BMC Res Notes. 2018 Feb 13;11:122. doi: 10.1186/s13104-018-3220-1 (PMC5809889; doi:10.1186/s13104-018-3220-1)
Supplement: Supplementary file 1 — Additional file 1. Supplemental descriptive and inferential statistics. Analyses conducted in order to examine the association between patient age, weight, sex, and malignancies of the spleen and liver. [file 13104_2018_3220_MOESM1_ESM.docx]

**Results**

**Introduction**

A series of analyses were conducted in order to examine the association between patient age, weight, sex, and malignancies of the spleen and liver. Independent-samples *t*-tests were conducted in order examine the associations with age and weight, and with chi-square analyses conducted with patient sex.

**Descriptive Statistics**

Table 1 presents some of the results of the descriptive statistics conducted on these data. With respect to age, mean age was found to be substantially higher among patients with no malignancies in all three cases. With respect to weight, mean weight was found to be slightly higher in cases where malignancies were present.

Table 1

*Age in Months and Weight in Kilograms by Malignancies*

Measure *N* Mean *SD*

*Age*

*Spleen Malignancy*

No 42 127.61 22.59

Yes 83 118.86 25.35

*Liver Malignancy*

No 57 124.24 24.43

Yes 68 119.48 24.97

*Both Lesions Benign or Both Lesions Malignant*

Benign 34 125.24 23.77

Malignant 60 117.80 25.39

*Weight*

*Spleen Malignancy*

No 42 25.97 12.09

Yes 83 28.80 11.21

*Liver Malignancy*

No 57 27.48 11.00

Yes 68 28.20 12.08

*Both Lesions Benign or Both Lesions Malignant*

Benign 34 27.77 11.08

Malignant 60 27.96 12.14

**Inferential Tests**

First, a series of independent-samples *t*-tests were conducted in order to determine whether there were significant differences in patient age on the basis of malignancies. With regard to spleen malignancies, Levene's test of the equality of variances was not found to achieve statistical significance, indicating that the assumption of the equality of variances was not violated in this analysis, *F* = .72, *p* = .399. The *t*-test conducted failed to achieve statistical significance, indicating that there was no significant difference in age on the basis of spleen malignancies, *t*(123) = 1.88, *p* = .063. Statistical significance was also not found in the Levene's test conducted on the analysis with liver malignancies, *F* = .03, *p* = .866. A significant difference in age was not found on the basis of liver malignancies, *t*(123) = 1.07, *p* = .285. With respect to whether patients had a malignancy in either the liver or spleen, Levene's test was not found to achieve statistical significance, *F* = .16, *p* = .690. The *t*-test conducted also failed to achieve statistical significance, *t*(123) = 1.69, *p* = .093.

The following set of analyses consisted of a series of independent-samples *t*-tests conducted with patient weight. First, in the analysis focusing upon spleen malignancies, Levene's test was not significant, *F* = .52, *p* = .474, with the *t*-test also not found to achieve statistical significance, *t*(121) = -1.29, *p* = .200. Similar results were found in the analysis conducted with liver malignancies, with neither Levene's test found to achieve significance, *F* = .69, *p* = .409, nor the *t*-test, *t*(121) = -.35, *p* = .730. Statistical significance was also not found in the Levene's test in the analysis conducted with whether patients had a malignancy in either organ, *F* = .82, *p* = .366, or in the *t*-test, *t*(121) = -.09, *p* = .930.

The final set of analyses conducted consisted of a series of chi-square analyses conducted with sex. No significant association was found between sex and spleen malignancies, χ^2^(3) = 7.01, *p* = .07, Cramer's *V* = .24. There was no significant association found with liver malignancies, χ^2^(3) = 1.52, *p* = .801, or whether patients had malignancies in both organs, χ^2^(3) = 3.12, *p* = .339. Table 2 reports the results of the crosstabulation between sex and spleen malignancies.

Table 2

*Crosstabulation of Sex and Spleen Malignancies*

Sex No Yes Total

FI 0 1 1

FS 15 44 59

MI 7 4 11

MN 20 34 54

Total 42 83 125
